# Inflammation and heterogeneity in synucleinopathies

**DOI:** 10.3389/fimmu.2024.1432342

**Published:** 2024-08-30

**Authors:** Antoine Freuchet, Anaëlle Pinçon, Alessandro Sette, Cecilia S. Lindestam Arlehamn

**Affiliations:** ^1^ Center for Autoimmunity and Inflammation, La Jolla Institute for Immunology, San Diego, CA, United States; ^2^ Aligning Science Across Parkinson’s (ASAP) Collaborative Research Network, Chevy Chase, MD, United States; ^3^ Master de Biologie, Ecole Normale Superieure de Lyon, University of Lyon, Lyon, France; ^4^ Department of Medicine, University of California, San Diego, San Diego, CA, United States

**Keywords:** neuroinflammation, neurodegeneration, Parkinson’s disease, dementia with Lewy bodies, multiple system atrophy, immunity, sex-based differences

## Abstract

Neurodegenerative diseases represent a huge healthcare challenge which is predicted to increase with an aging population. Synucleinopathies, including Parkinson’s disease (PD), dementia with Lewy bodies (DLB), and multiple system atrophy (MSA), present complex challenges in understanding their onset and progression. They are characterized by the abnormal aggregation of α-synuclein in the brain leading to neurodegeneration. Accumulating evidence supports the existence of distinct subtypes based on the site of α-synuclein aggregation initiation, genetics, and, more recently, neuroinflammation. Mediated by both central nervous system-resident cells, peripheral immune cells, and gut dysbiosis, neuroinflammation appears as a key process in the onset and progression of neuronal loss. Sex-based differences add another layer of complexity to synucleinopathies, influencing disease prevalence - with a known higher incidence of PD in males compared to females – as well as phenotype and immune responses. Biological sex affects neuroinflammatory pathways and the immune response, suggesting the need for sex-specific therapeutic strategies and biomarker identification. Here, we review the heterogeneity of synucleinopathies, describing the etiology, the mechanisms by which the inflammatory processes contribute to the pathology, and the consideration of sex-based differences to highlight the need for personalized therapeutics.

## 1 Introduction

In this review, we discuss the roles of inflammation mediated by central nervous system (CNS)-resident cells, gut dysbiosis, and peripheral T and B cells in synucleinopathies. We also highlight the importance of considering sex-based differences in future studies, and discuss potential therapeutic approaches.

The incidence of neurodegenerative diseases, such as Alzheimer’s and Parkinson’s disease (PD), is increasing in the US as life expectancy and the elderly population rise (1). Constituting the second most common neurodegenerative disease in the elderly population after Alzheimer’s disease, PD affects more than 10 million individuals, a number expected to double over the next 30 years (2, 3). These disorders are typically characterized by the abnormal aggregation of misfolded proteins in the central nervous system, namely accumulation of hyperphosphorylated tau and beta-amyloid (Aβ) plaques in Alzheimer’s disease and α-synuclein (α-syn) in synucleinopathies like PD (4). Lewy body diseases such as PD and DLB, are characterized by α-syn aggregation in Lewy bodies (LBs) and Lewy neurites (LNs) in neuronal cells. In contrast, in MSA α-syn first accumulates in glial cytoplasmic inclusions (GCIs) in oligodendrocytes, interfering with oligodendrocyte survival and neuronal support (5).

While the precise functions of α-syn are not yet entirely known, its pre-synaptic localization and association with synaptic vesicles, indicates a likely role in regulating neurotransmitter release, synaptic function, plasticity (6, 7) and its ability to bind to lipid membrane (8). Misfolded and aggregated α-syn in neuronal and glial cells, is associated with the development of synucleinopathies, such as PD, Dementia with Lewy Bodies (DLB), and multiple system atrophy (MSA), and thus understanding the transition from normal to abnormal α-syn is crucial for our understanding the development of synucleinopathies. Incidence increases sharply with age, and men are more affected than women (9, 10). Given the heterogeneity observed and described across studies within synucleinopathies, ongoing efforts are focusing on stratifying patients. Recent evidence suggests sex-based differences in immune responses both at steady state and in autoimmune diseases (11), though studies on sex differences in synucleinopathies are limited (12, 13) despite known sex bias, clinical, and symptomatic differences, as observed in PD (14). Thus, taking biological sex as a subdividing variable has the potential to highlight crucial differences and hint towards new therapeutic approaches.

Although synucleinopathies all involve α-syn aggregation and neuronal loss, they exhibit differences in clinical and pathological characteristics. PD typically manifests with a long non-motor prodromal phase followed by motor parkinsonism, including rigidity, bradykinesia, and resting tremor (15). During the prodromal phase, PD patients often experience gastrointestinal disturbances, consistent with the emerging gut-brain theory suggesting that α-syn pathology initiates in the gut before spreading to the brain (16). Rapid eye movement (REM) sleep behavior disorder (RBD) is also a common prodromal non-motor symptom across synucleinopathies, occurring in 30-70% of PD patients, 70-80% of DLB patients, and 70-90% of MSA patients (17). In later stages, most PD patients develop dementia, progressing to Parkinson’s disease dementia (PDD). DLB shares clinical characteristics with PD, but typically dementia presents itself either preceding or within a year of parkinsonism symptoms (18). Conversely, MSA does not involve dementia and is primarily characterized by autonomic nervous system dysfunction, including urinary incontinence, in addition to cerebellar ataxia and parkinsonism (19). MSA is notably the most aggressive synucleinopathy and is associated with a more acute inflammatory response, suggesting that enhanced inflammation contributes to a more aggressive clinical course.

Neuroinflammation is a common feature of neurodegenerative diseases (20). In a physiological context, the inflammatory response, primarily mediated by microglia and astrocytes within the CNS, is essential for maintaining homeostasis by promoting tissue repair and clearing cellular debris. However, neuroinflammation also plays a critical role in disease pathogenesis (21). In synucleinopathies, activation of CNS-resident microglia and astrocytes leads to the expression of pro-inflammatory cytokines, which can directly induce neurotoxicity, disrupt the blood-brain barrier (BBB) (22), or recruit immune cells from the periphery to the CNS through the secretion of chemokines (23). Chronic inflammatory responses, characteristic of neurodegenerative diseases like synucleinopathies, exacerbate neuronal loss, leading to further neuroinflammation in a vicious cycle (21). The emerging gut-brain theory invokes a peripheral origin of inflammation, with intestinal dysbiosis initiating early α-syn aggregation and inflammation in the gut before spreading to the brain in PD (24).

Mediated by both CNS-resident cells and the periphery, inflammation appears to be a key process in the onset and progression of neurodegeneration in synucleinopathies. Understanding how inflammatory responses are induced within the CNS and how these responses contribute to neurodegeneration will aid in developing new therapies targeting inflammation to reverse or slow disease progression.

## 2 Etiology of synucleinopathies

Although a comprehensive understanding of the onset of synucleinopathies remains elusive, accumulating evidence indicates the existence of at least two disease subtypes based on the site of α-syn aggregation initiation: brain-first or body-first. In both subtypes, misfolded α-syn propagates from cell to cell in a prion-like manner (25), facilitating disease progression (26). Thus, α-syn aggregation represents a hallmark of synucleinopathies depending on where inclusions are found. In PD and DLB, α-syn spreads from neuron to neuron (i.e., Lewy bodies and Lewy neurites) and also involves astroglial cells (27) whereas in MSA, α-syn primarily accumulates in oligodendrocytes (i.e., glial cytoplasmic inclusions) (5). Inflammation has been found mainly in PD, with fewer studies in MSA and DLB (20). It could suggest a role of the immune system in the initiation of the disease, although it can be a consequence as well. The differences in initiation sites and underlying mechanisms contribute to variations in the kinetics of symptom development, emphasizing the importance of distinguishing between these subtypes to gain deeper insights into the underlying biology and to develop targeted therapies.

### 2.1 Body-first

Neurodegenerative diseases have traditionally been investigated within the CNS, but there is mounting evidence suggesting the involvement of the enteric nervous system (ENS) as the initiator of CNS diseases, particularly in the body-first subtype. Braak et al. proposed that α-syn diseases could originate in the periphery, spreading from the gut to the brain via the vagus nerve in a prion-like fashion (28), through a disease progression pattern of several stages of increasing severity. During the early prodromal phase, the vagus nerve and the olfactory bulb are affected, correlating with the early presence of gastrointestinal disturbances such as constipation and olfactory loss. The body-first disease subtype is strongly associated with RBD, with studies showing that almost all RBD patients develop synucleinopathies (PD, DLB, or MSA) (17, 29, 30). The progression to later stages affects dopaminergic neurons of the substantia nigra, correlating with motor symptoms, and further spread of pathology affects cortical structures, leading to dementia.

The hypothesis is that abnormal microbiota results in increased gut permeability and accumulation of aggregated α-syn in the gut, which then spreads through the vagus nerve to the brain. Increasing data support this gut-brain theory; α-syn aggregates are detected early in the ENS of PD patients (31), and inoculation of α-syn fibrils into the gut of aged mice induces CNS pathology (32). Intestinal α-syn aggregation is attributed to changes in gut microbial composition, particularly microbial dysbiosis associated with aging or a poor diet (33). Most PD patients exhibit altered microbiota, characterized by decreased anti-inflammatory short-chain fatty acid (SCFA)-producing bacteria and increased gut layer-degrading bacteria (34, 35). A meta-analysis confirmed gut microbiome alterations in PD, potentially leading to increased pro-inflammatory status and gastrointestinal symptoms (36). This results in local inflammatory reactions and increased intestinal permeability, exposing the intestinal neural plexus to toxins like pesticides or lipopolysaccharide (LPS), which promote abnormal α-syn aggregation. In the presence of LPS, α-syn accumulates in a specific fibrillar form that can self-propagate and spread between interconnected neurons of the vagus nerve in a prion-like manner (26). Increased intestinal permeability also leads to leakage of inflammatory factors from the gut, causing systemic inflammatory responses that impair the BBB and facilitate inflammatory mediator uptake into the brain. Consequently, α-syn accumulation in the gut may trigger pro-inflammatory glial responses and CNS neuroinflammation.

Recently, the functional link between gut microbiota and neuroinflammation was demonstrated in a murine model of PD overexpressing α-syn. Colonization of these mice with microbiota from PD patients induced α-syn aggregation, microglial activation, and motor deficits, unlike in germ-free mice (37). In line with those results, a prebiotic treatment in a murine model of PD reduces the severity of the disease (38). A study by Garretti et al. demonstrated that immunization with a specific ⍺-syn epitope (31–45) in a transgenic mice model carrying the HLA allele DRB1*15:01 can trigger gut inflammation in a CD4 T cell-dependent manner (39). Overall, this data supports the notion that microbial dysbiosis in PD may initiate the inflammatory process and underlying neurodegeneration.

Several lines of evidence suggest that the body-first model might be applicable to MSA as well. Studies have linked pro-inflammatory microbiota to increased gut permeability in MSA patients, supporting a gut-brain interaction (40, 41). However, it remains uncertain whether this model might apply to DLB, as dementia precedes motor symptoms, contradicting the Braak model.

### 2.2 Brain-first

The concept of a brain-first subtype emerged from several studies highlighting neuropathological events not conforming to the proposed Braak staging (42–44). It has been proposed that α-syn pathology might primarily originate within the CNS, likely rostral to the substantia nigra pars compacta, before spreading to affect the autonomic nervous system (45). Moreover, RBD in PD patients has been suggested as a discriminative marker between body-first and brain-first. A comprehensive study employing multimodal imaging revealed distinct patterns: PD patients with RBD initially exhibit cardiac ^123^I-metaiodobenzylguanidine (MIBG; measuring cardiac innervation), and ^11^C-colonic donepezil (measuring colon innervation) signal loss, followed by a decrease in putaminal FDOPA (measuring nigrostriatal dopamine storage capacity) uptake, indicative of a body-first subtype. Conversely, PD patients without RBD display a different sequence: primary putaminal FDOPA uptake loss followed by secondary cardiac MIBG and ^11^C-donepezil signal loss, suggesting a brain-first subtype (46). Other differences associated with the presence of RBD include varied motor symptom patterns, more frequent and severe constipation, potential urinary symptom increases, and heightened olfactory dysfunction (47). These findings confirm the existence of at least two PD subtypes and underscore the potential utility of pre-motor RBD as a diagnostic marker.

Interestingly, genetic variants may be associated with either brain- or body-first trajectories. The *LRRK2* variant, for instance, is associated with lower RBD prevalence across studies and nearly normal cardiac MIBG signal, resembling the brain-first subtype. Conversely, the *SNCA* variant exhibits a slightly higher RBD incidence, indicative of a body-first subtype similar to the pathogenic *GBA* variant (47). It is now widely recognized that PD patients constitute a highly heterogeneous population, and identifying different synucleinopathy initiation sites can aid in subtype discrimination.

## 3 Resident cells of the CNS as players of the inflammation

### 3.1 Complex role of microglia

Microglia are the most abundant cells involved in innate immune responses within the CNS. Recent advancements from transcriptomic, morphological, metabolomic, epigenetic, and proteomic studies have revealed the heterogeneity of microglia under steady-state conditions and in disease states (48). Microglia serve as critical sensors in the CNS, responding to various stimuli such as presence of apoptotic cells, debris, and toxic proteins. Extensive microgliosis, characterized by highly activated microglia, has been observed in post-mortem brains in regions containing α-syn Lewy bodies in PD (49) and regions with α-syn glial cytoplasmic inclusions in MSA (50). While initially beneficial for phagocytosing α-syn aggregates, microglia-mediated inflammatory responses have also been shown to contribute to neurodegeneration. Aggregated α-syn activates toll-like receptors (TLRs) TLR2 and TLR4 on microglia, triggering a pro-inflammatory signaling cascade mediated by NFκB and p38 MAPK pathways. This cascade leads to the secretion of pro-inflammatory cytokines TNF-α, β, IL-1β, IL-6, and IL-1α, known to exert neurotoxic effects (51, 52). Additionally, TLR activation by α-syn induces the release of reactive oxygen species (ROS) and nitric oxide (NO), causing neuronal mitochondrial dysfunction, DNA damage, and subsequent neurotoxicity (53). Thus, α-syn acts as a pathogen-associated molecular pattern (PAMP) or a damage-associated molecular pattern (DAMP), promoting microglia-mediated inflammation that chronically supports neurodegeneration. Furthermore, activated microglia contribute to increased BBB permeability by releasing cytokines, upregulating adhesion molecules, and phagocytizing astrocyte end-feet, which normally maintain BBB integrity. This facilitates the invasion of peripheral immune cells into the CNS, amplifying neuroinflammation (54). Additionally, as antigen-presenting cells (APCs), activated microglia play a pivotal role in initiating T cell-mediated immune responses.

Accumulating evidence suggests that microglia play a crucial role in disease initiation: α-syn aggregation induces reactive microgliosis months before neuronal cell death in PD, implying that microglia may not only exacerbate but also initiate neurodegeneration (55). In contrast, there is no substantial evidence supporting significant microglial activation in DLB: post-mortem brain analyses have failed to demonstrate microgliosis, suggesting that inflammation may be limited, at least at the end-stage of the disease (56, 57).

### 3.2 Emerging inflammatory role of astrocytes

Astrocytes, the most abundant resident glial cells in the CNS, perform crucial homeostatic functions including synaptic and BBB maintenance, elimination of excess synaptic connections, and supplying neurons with vital metabolites (58). While microglia have long been considered the primary immune effector cells of the CNS, it is now recognized that astrocytes also play crucial roles in innate immunity, and are implicated in neuroinflammation associated with neurodegenerative processes (59). Elevated astrogliosis, characterized by the activation and accumulation of astrocytes, is observed in response to neurodegeneration in the CNS (50).

Reactive astrocytes encompass two distinct types, termed A1 and A2 (60). A1 astrocytes lose typical astrocyte functions and acquire neurotoxic properties, while A2 astrocytes express neurotrophic factors and confer neuroprotection (61). In synucleinopathies, α-syn aggregates trigger astrocyte activation and differentiation into an A1 phenotype. Indeed, postmortem brains from human neurodegenerative diseases, including PD, exhibit A1 astrocytes expressing Glial Fibrillary Acidic Protein (GFAP+) (62). Additionally, GFAP+ astrocytes colocalize with GCIs in models of MSA (63), although their presence in DLB has been reported in only one study to date (64).

The α-syn-mediated activation of astrocytes, akin to microglia, is facilitated by TLR2 and TLR4 (52, 65). This activation initiates downstream signaling cascades, resulting in the release of pro-inflammatory cytokines (IL-1β, IL-1α, TNF-α) and NO, contributing to neurotoxicity (60). The classical complement component C1q enhances synaptic degeneration, while CCL5/CX3CL1 chemokines recruit reactive microglia (65). It has been demonstrated that microglia can induce A1 neurotoxic reactive astrocytes, and blocking this microglial-mediated conversion is neuroprotective. Furthermore, A1 astrocytes may secrete soluble neurotoxins such as D-serine, which rapidly kills neurons (66). Although controversial, recent studies suggest that astrocytes may act as APCs, implicating them in initiating adaptive T cell immunity (67). Normally, astrocytes contribute to BBB integrity through their end-feet and production of supportive molecules for endothelial cells (68). However, microglial phagocytosis of end-feet and the presence of α-syn aggregates impair astrocyte functions, contributing to BBB leakage and facilitating immune cell infiltration and inflammatory molecule entry from the periphery, further amplifying neuroinflammation (68, 69).

**Figure 1:**
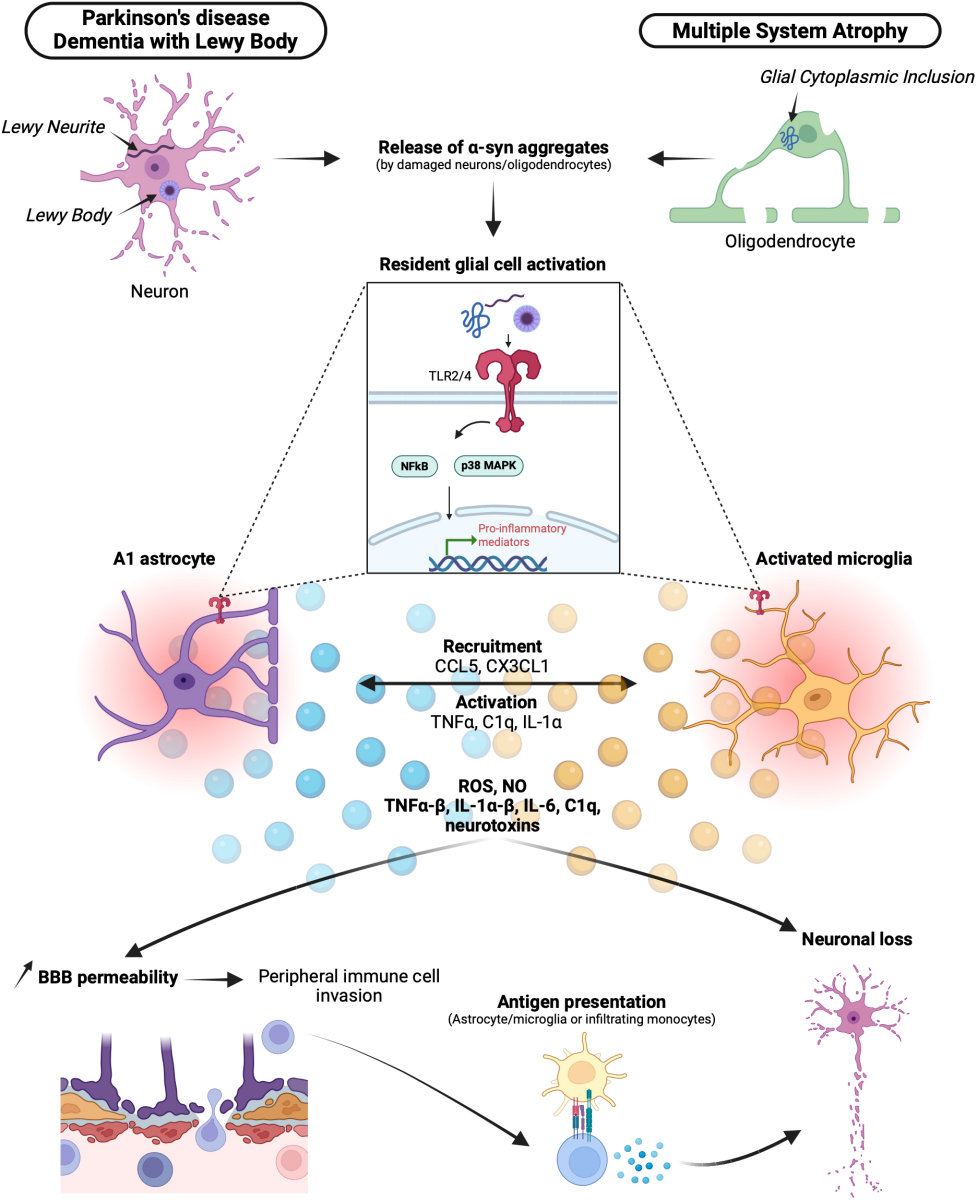
Brain resident cells in synucleinopathies and neuroinflammation. In synucleinopathies, α-syn abnormally accumulates in Lewy bodies (LBs) or Lewy neurites (LNs) for Lewy body diseases DLB and PD or in glial cytoplasmic inclusions (GCIs) for MSA. α-syn aggregates are released by damaged neurons and oligodendrocytes and recognized by resident astrocytes and microglia via Toll-like receptor 2 (TLR2) and Toll-like receptor 4 (TLR4). Activation of TLRs by α-syn leads to the differentiation of activated pro-inflammatory A1 astrocytes and activated microglia. Through a pro-inflammatory signaling cascade mediated by NFκB and p38 MAPK, A1 astrocytes and activated microglia express several chemokines, cytokines, neurotoxins, reactive oxygen species (ROS), and nitric oxide (NO). This pro-inflammatory milieu contributes to neuronal loss. Additionally, with the action of activated microglia phagocytizing astrocytes, the integrity of the blood-brain barrier (BBB) is compromised, allowing the invasion of peripheral mediators and immune cells. Among them, T cells can be activated via the recognition of a specific major histocompatibility complex (MHC)-peptide presented by astrocytes, microglia, or infiltrating monocytes, ultimately leading to neuronal loss. α-syn, α-synuclein; DLB, dementia with Lewy bodies; PD, Parkinson’s disease; MSA, multiple sclerosis atrophy; LBs, Lewy bodies; LNs, Lewy neurites; GCIs, Glial cytoplasmic inclusions; TLRs (2-4), toll-like receptors; ROS, reactive oxygen species; NO, nitric oxide; BBB, blood-brain-barrier.

In summary, CNS-resident cells, microglia and astrocytes, play critical roles in initiating and amplifying the inflammatory process in synucleinopathies. This leads to the production of neurotoxic mediators, neuronal degeneration, recruitment of peripheral immune cells, and potentiation of CNS inflammation, contributing to disease progression (**Figure 1**).

## 4 Involvement of peripheral immune cells in synucleinopathies

**Figure 2:**
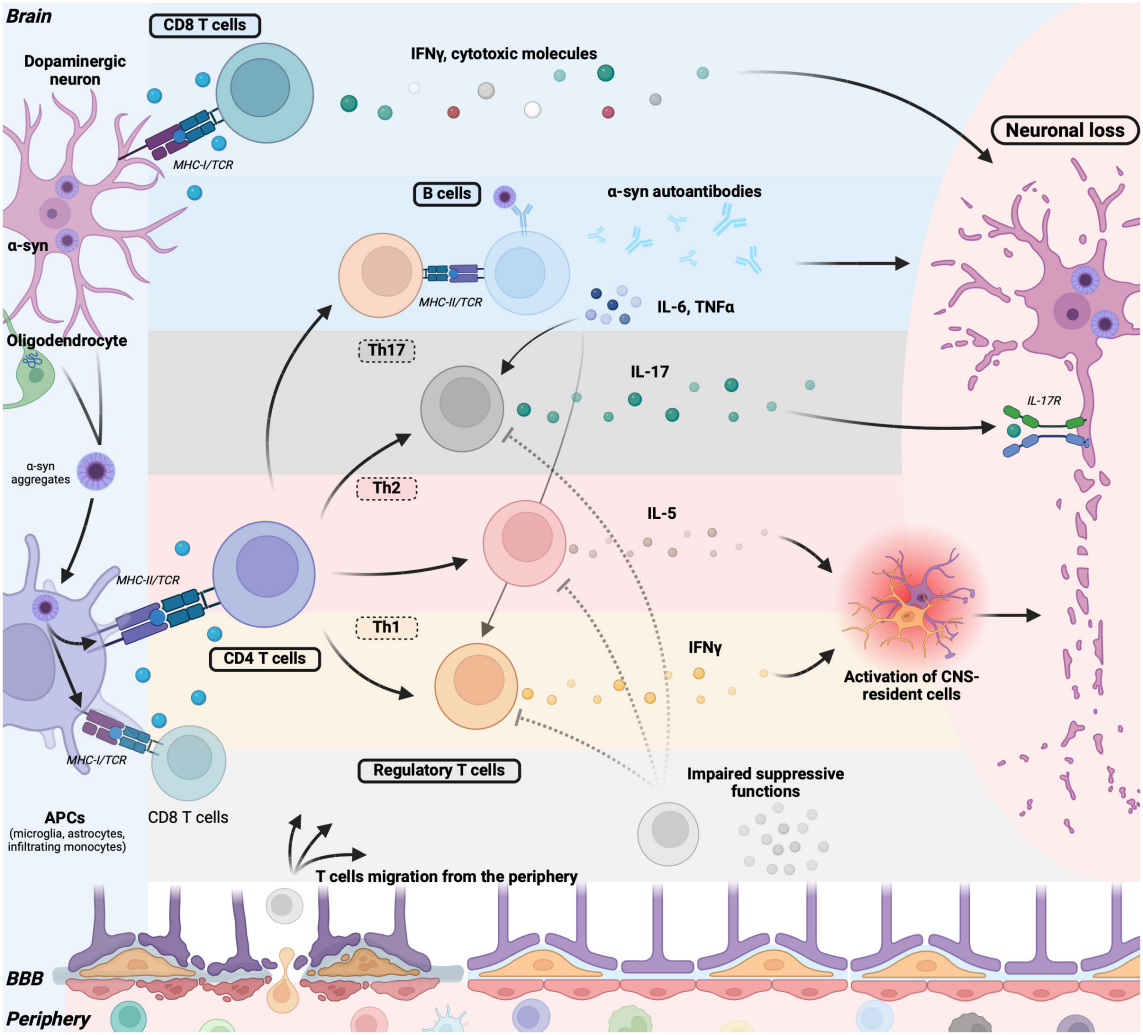
Adaptative immune cells contribute to neurodegeneration in synucleinopathies. Pathogenic α-syn aggregates in neurons (PD, DLB) or in oligodendrocytes (MSA) are released and captured by antigen-presenting cells (APCs) such as microglia, astrocytes, and infiltrating monocytes. These APCs process and present α-syn peptides on their major histocompatibility complex (MHC) molecules. Alternatively, neurons can directly present α-syn peptides on their MHC class I molecule. Upon TCR triggering, CD8+ T cells secrete IFNγ or cytotoxic granules (Granzymes, perforin), inducing neuronal death. B cells recognize α-syn through their BCR and, with the aid of T helper cells, mature into plasma cells producing α-syn autoantibodies, potentially contributing to neuronal damage. B cells also release pro-inflammatory cytokines such as TNFα and IL-6, promoting T cell differentiation into Th1 and Th17 cells, thereby contributing to T cell-mediated inflammation. Following MHC-II/TCR interaction, CD4+ T cells further differentiate into Th17 cells, producing IL-17 that directly promotes neuronal loss via neuronal IL-17 receptors. CD4+ T cells can also differentiate into Th1 or Th2 cells, secreting IFN-γ and IL-5, respectively, further activating CNS-resident microglia and astrocytes, thereby promoting a deleterious inflammatory environment. Regulatory T cells in synucleinopathies exhibit impaired suppressive functions, failing to counteract T cell-mediated inflammation. Lastly, the BBB is compromised in patients, allowing the migration of immune cells and mediators into the brain, perpetuating the pro-inflammatory loop. α-syn, α-synuclein; DLB, dementia with Lewy bodies; PD, Parkinson’s disease; MSA, multiple sclerosis atrophy; MHCI-II, major histocompatibility complexes I-II; TCR, T-cell receptor; APCs, antigen-presenting cells; BCR, B-cell receptor; BBB, Blood brain barrier; CNS, central nervous system.

The debate surrounding whether immune cells from the periphery can traverse the BBB has persisted for years. Traditionally, the brain was viewed as an immunologically privileged organ. However, recent insights into the breakdown of the BBB during acute or chronic inflammation, which permits peripheral molecules and immune cells to access the CNS (22), along with the discovery of a lymphatic system within the CNS (70), have nuanced this perspective. Consequently, while studies have primarily focused on inflammation mediated by CNS-resident cells, emerging research highlights the crucial roles of inflammation mediated by T and B cells (**Figure 2**), and myeloid cells in synucleinopathies.

### 4.1 T cells

The first indications of T cell involvement in synucleinopathies stem from the high expression of major histocompatibility complex II (MHC-II) in the CNS on microglia and astrocytes, and cytokine production by CNS-resident cells implicated in T cell differentiation and recruitment (67, 71, 72). MHC-II, a cell surface protein on APCs, binds peptides derived from antigens like abnormal ⍺-syn and presents them to CD4 T cells, facilitating their differentiation and infiltration to the CNS. Notably, elevated MHC-II expression has been observed on CNS microglia and astrocytes in postmortem brains of individuals with PD and MSA, which may provide a link with adaptive T cell immunity (67, 71). Elevated levels of IL-6—a pivotal cytokine involved in CD4 T cell differentiation into pro-inflammatory Th17 effector cells—have been detected in DLB patients (73). These clues pointing to T cell involvement in synucleinopathies are further supported by the presence of T cell infiltrates surrounding α-synuclein LBs or GCIs in the substantia nigra of postmortem PD and MSA tissues (71, 74), as well as in cortical areas surrounding LBs in postmortem DLB tissues (57). Studies have demonstrated that T cells can recognize a specific set of peptides derived from ⍺-syn, driving helper CD4 and cytotoxic CD8 T cell responses in PD patients (75–79). Consequently, CD4 T cells recognize α-syn antigens presented by MHC-II on APCs, while CD8 T cells recognize α-syn derived peptides presented by MHC-I, potentially by neurons (80) which remains to be proven. This T cell activation may mediate both direct and indirect neuronal damage (81).

The involvement of CD4 T cells in neurodegeneration is supported by both *in vivo* studies and *in vitro* culture systems. Deficiency in CD4 T cells results in a significant attenuation of neurodegeneration (74). Pro-inflammatory Th1/Th2 CD4 T cells may contribute to neuronal loss by secreting cytokines such as IFNγ and IL-5, activating CNS resident cells, and mediating neuroinflammation and tissue damage (71, 76, 82). Moreover, Th17 CD4 T cells can directly damage neurons by secreting IL-17, which is neurotoxic when recognized by IL-17R on neurons (83). At the level of alterations in CD4 T cell subpopulations (84), some studies pointed out a decreased frequency of CD4 T cells (85), Th2 or Th17 (86) in PD treated compared to healthy controls (HC), others an increase (87, 88) or no difference (89, 90). Looking at antigen-specific T cells, we have shown the existence of ⍺-syn reactive T cells in the blood, which exhibit a higher magnitude of response in PD than HC (75, 76). Additionally, we demonstrated a higher T cell reactivity to ⍺-syn within 10 years since diagnosis (76), demonstrating that antigen-specific T cells can be used as a biomarker in PD.

Aligned with the gut-brain theory in PD, it has been proposed that the development of α-syn-specific T cell responses initially occurs in the gut before extending to the brain. Local antigen-presenting cells, such as mucosal dendritic cells, recognize α-syn aggregates, migrate to the mesenteric lymph node, and present α-syn antigens to CD4 T cells via MHC-II, triggering differentiation into pro-inflammatory α-syn-specific CD4 T cells Th1 and Th17. Subsequently, these pro-inflammatory T cells migrate to the brain, promoting neuroinflammation, years after α-syn has reached the brain (91).

Discordant results regarding regulatory T cells (Tregs) frequency compared to healthy controls are found in the literature. Some studies report an increase, decrease, or no change in Treg frequency (72, 92). As Tregs primarily suppress T cell proliferation and cytokine production, impaired function leads to immune system dysregulation and inflammation (93, 94). Tregs from PD patients exhibit decreased suppressive ability over T cell proliferation, and CD4 T cells from patients produce heightened levels of IFNγ and TNF in response to polyclonal stimulation compared to HC, complicating immune response regulation (86). Other regulatory cell populations, such as Tr1 cells, IL-10-producing CD8 Tregs, and tolerogenic dendritic cells, show decreased frequency in PD patients (90). Overall, the regulatory compartment in PD patients appears altered, suggesting the potential development of therapies focusing on restoring regulatory functions. Indeed, *ex vivo* expansion of Tregs from PD patients enhances suppressive function and demonstrates a stronger Treg gene signature (94), while intra-striatal co-transplantation of Treg cells with human-induced pluripotent stem cell-derived midbrain dopaminergic neurons protects grafted cells and improves therapeutic outcomes in rodent PD models (95).

The role of CD8 T cells remains unclear, with studies showing their potential to induce neuronal death in an IFNγ-dependent manner (96) or via cytotoxic functions (77, 97), yet genetic knockout of CD8 does not impact CNS myeloid activation (98). Conflicting results regarding their frequency in peripheral blood from PD have also been reported (72, 84). A neuropathological study on PD and DLB post-mortem brains demonstrated an early infiltration of CD8 T cells, but not CD4 T cells, in the SNpc. Interestingly, the recruitment of CD8 T cells precedes ⍺-syn aggregation (96), which is in contrast to what was observed in multiple sclerosis (99) or Type 1 diabetes (100). Further work is thus needed to better characterize CD8 T cells to understand their part in synucleinopathies.

### 4.2 B cells

Cell-mediated immunity orchestrated by T cells has been extensively studied in synucleinopathies, less attention has been given to humoral immunity mediated by B cells. However, B cells play crucial roles beyond antibody production, including antigen presentation to T cells and cytokine secretion, contributing significantly to neuroinflammation.

Upon antigen recognition by their B-cell receptor (BCR), B cells differentiate into plasma cells and secrete specific antibodies, which have been detected in the serum and CSF of patients with synucleinopathies (101). Notably, α-syn-specific autoantibodies are localized in Lewy bodies in postmortem analyses of PD patients, underscoring their specificity for α-syn (102). Moreover, microglia in PD patients exhibit abnormally high expression of Fc gamma receptors (FcγRs) (101), suggesting that the infiltration of these autoantibodies into the CNS contributes significantly to neuroinflammation, although the exact roles of α-syn-specific autoantibodies remain to be fully elucidated (101).

In addition to antibody production, B cells modulate immune responses through antibody-independent functions, such as cytokine secretion. Recent evidence indicates that pro-inflammatory B cells producing TNFα and IL-6 are increased, while anti-inflammatory IL-10-producing B cells are decreased in PD patients, reflecting a pro-inflammatory shift in B-cell cytokine responses (103). These pro-inflammatory B cells can activate Th1 and Th17 cells, further contributing to T-cell-mediated inflammation (104) Moreover, B cells can act as APCs to CD4 T cells via MHC-II, enhancing T cell activation. Consistent with this, up-regulation of MHC-II genes has been observed in B cells of PD patients, indicating enhanced antigen presentation capacity (105). Conversely, a decrease in MHC-II expression on B cells has been reported in DLB, suggesting reduced B cell activation and potentially diminished humoral adaptive immunity in this disease (73). Despite the need for further clarification regarding the diverse roles of B cells, their involvement in inflammation and neurodegeneration in synucleinopathies is evident.

### 4.3 Myeloid cells

The investigation of myeloid cells (except for microglia) in synucleinopathies has been mainly focused in PD. However, in a MSA mice model, the depletion of myeloid cells using CSF1R inhibitor (PLX5622) surprisingly improved overall survival with a delayed onset and reduced inflammation, but animals presented severe impaired motor functions, synaptic signaling, and neuronal circuitries (106). In a follow-up investigation, it has been shown that the PLX5622-induced impaired motor functions were potentially linked to a shift in the neuronal balance with an increased inhibitory connectivity (107). As PLX5622 acts on all CSF1R expressing cells, its action is not restricted to microglia, thus the dual result can be due to the depletion of monocytes or Border Associated Macrophages (BAM).

Monocytes can in the context of PD enter the CNS through the expression of CCR2 (108), differentiate into macrophages with different functions from microglia (109, 110). A study has shown the ability of mouse CD11c^+^ cells activated by ⍺-syn to circulate from the brain to the gut providing new insight on the disease propagation (111). The entry of monocytes into the CNS in a mice model of PD is associated with inflammation and neurodegeneration (108) showing the promise of therapeutic target. In addition, myeloid cells can act as APC and activate T cells, further participating in the disease progression. In this context, Schonhoff et al. have demonstrated that BAM are increased by ⍺-syn in a mice model. Furthermore, BAM, but not microglia as initially thought, are responsible for CD3^+^ T cells activation with evidence of interactions in the perivascular space of PD brains (112). At the transcriptome level, blood monocytes from early diagnosed PD have specific signatures compared to HC. Interestingly, differences were stronger when focusing on females with genes enriched in pro-inflammatory pathways such as Natural killer cell cytotoxic and Antigen processing and presentation (113). This was confirmed in a study part of The Myeloid cells in Neurodegenerative Diseases (MyND) initiative. BulkRNA-seq and Single-cell RNAseq on CD14^+^ monocytes from PD blood supported transcriptomic alterations, specifically in the mitochondrial and proteasome with a higher pro-inflammatory signature in CD14^+^CD16^+^ intermediate monocytes (110).

CD163, restricted to the monocytes and macrophages lineage, has been investigated in several studies as its expression has been found in PD brains (114), on peripheral blood monocytes of early diagnosed PD (115) as well as RBD patients (prodromal patients) (116). Its expression is sought to be protective especially in women. Indeed, female CD163KO mice present increased dopaminergic neuronal loss, whereas in male mice, its deletion leads to similar T cell activation without SN loss (117). Similarly, investigation of soluble CD163 (sCD163) highlighted a sex specific difference in females compared to males. Serum levels were only higher in PD females compared to HC, and in CSF sCD163 levels correlated with immune system activation markers and inversely with cognitive scores. The authors stated that ⍺-syn activates macrophages which induces CD163 shedding and increases ⍺-syn clearance (118). Thus, sCD163 represents a potential biomarker of PD in females.

Altogether, the diverse functions, heterogeneity, and complexity of myeloid cells in synucleinopathies represent a challenge currently being tackled, but holds the promise of effective therapies to limit the disease progression and T cell activation.

## 5 Sex-based differences further stratify synucleinopathies

The focus on sex-based differences in diseases, especially those with an autoimmune component, has intensified in recent years. Sex differences are evident in synucleinopathies (**Table 1**), particularly in PD, where men show a higher prevalence and incidence, while women experience greater mortality and require earlier professional help (119, 120). In PD, the phenotype, including the onset of symptoms, type of motor and non-motor symptoms, and levodopa bioavailability, differs between males and females. For instance, motor symptoms, kinetics, and severity follow distinct patterns. In comparison to men, women typically exhibit delayed clinical signs, less rigidity, and tremor as the initial symptom. They are more prone to postural instability and motor complications induced by levodopa medication. Non-motor symptoms are also more severe in women, encompassing fatigue, depression, restless legs, constipation, pain, loss of taste or smell, weight changes, and excessive sweating (121–125). Various factors such as socioeconomic status, genetics, environment, or gender bias may contribute to these sex-based differences.

**Table 1:** Sex-based differences in synucleinopathies.

	Synucleinopathy	Female	Male	Comments
**Epidemiology**	**PD**	Higher mortality Earlier professional help	Higher prevalence Higher incidence	Socioeconomic status, Genetics, Gender bias not studied
	**MSA/DLB**	Conflicting results		
**Motor symptoms**	**PD**	Higher pain More prone to postural instability More complications by levodopa medication	Earlier clinical signal More prone to rigidity More prone to tremor	
	**MSA**	More prone to present motor symptoms at onset	More prone to orthostatic intolerance More prone to early catheterization	Not fully overviewed
	**DLB**	Not thoroughly studied		
**Non-motor symptoms**	**PD**	More severe: fatigue, depression, restless legs, constipation, pain, loss of taste or smell, weight changes, and excessive sweating Protective role of estrogens in the brain	More severe cognition impairment Higher expression of *PINK1, SNCA* in SNc neurons Slightly higher rates of RBD	
	**DLB**	More prone to hallucinations Higher neuropsychiatric inventory	More prone to severe dementia Higher rates of antipsychotic use	
**Inflammation**	**PD**	Regulated microglia Anti-inflammatory astrocytes	Pro-inflammatory astrocytes	Sex-specific T cell reactivity not studied
	**MSA/DLB**	Not thoroughly studied		

Summary of sex-based differences in Parkinson's disease (PD), Multiple system atrophy (MSA) and Dementia with Lewy bodies (DLB) divided by epidemiology, motor and non-motor symptoms and inflammation. RBD, REM sleep behavior disorder; PINK1, PTEN-induced kinase 1; SCNA, Synuclein Alpha; SNc, Substantia nigra compacta.

Sex differences have been less explored in DLB and MSA, where the prevalence and incidence in men have shown conflicting results across studies. However, a few studies have identified different symptoms between males and females. For example, hallucinations are more prevalent in women with DLB, associated with older age and high neuropsychiatric inventory, whereas men present more severe dementia and higher rates of antipsychotic use (13, 126). In MSA, women are more likely to present motor symptoms at onset, while men exhibit a higher likelihood of orthostatic intolerance and early catheterization, contributing to a poorer survival rate (120). Further research focusing on sex differences in DLB and MSA is necessary to clearly define each subtype and identify biomarkers.

From a biological standpoint, early analyses have shown a correlation between the age of PD onset and the duration of fertile life, implicating a potential hormonal influence (127, 128). Estrogens play a protective role in PD, as the incidence in men and post-menopausal women is similar (129). The brain itself is influenced by biological sex, with anatomopathological differences observed between males and females with PD. Additionally, PD males tend to perform worse than females in global cognition, immediate verbal recall, and mental processing speed, while females exhibit less visuospatial function (130). The transcriptome of SNc dopamine neurons from males is associated with PD pathogenesis (*SNCA* and *PINK1*), whereas females show upregulation of genes involved in signal transduction and neuronal maturation (12, 131, 132).

Neuroinflammation is a significant component in synucleinopathies. Sex differences in the innate and adaptive responses in the periphery have been reviewed elsewhere (11). In the brain, estrogens play a protective role by modulating microglia responses to proinflammatory stimuli, while astrocytes from men upregulate proinflammatory cytokines compared to those from women, who express more anti-inflammatory cytokines (133, 134). Few studies focused on sex-based differences in inflammation in synucleinopathies. Among them, Mitra et al. observed in a rotenone-induced PD mouse model that males and females have different resident cell proportions in the SN. Males showed a decreased level of microglia and increased level of astrocytes compared to females (135). More recently, it has been shown that peripheral blood monocytes are differently activated in men and women PD patients, with a higher inflammatory profile and gene enrichment associated to IFNg stimulation in females (113). In addition, sCD163 serum and CSF levels is also specific to PD female (117, 118). Thus, segregating women and men when studying immune response to ⍺-syn remains to be investigated in PD.

A recent systematic review highlighting sex-based differences in synucleinopathies emphasized the necessity of designing future studies that subdivide males and females (120). Altogether, biological sex is an important factor to consider in stratifying synucleinopathies, offering new insights and potentially leading to more effective treatments.

## 6 Therapeutic perspectives to treat inflammation

It is now recognized that synucleinopathies are associated with an inflammatory component, prompting significant efforts towards developing inflammation-targeting strategies spanning from diagnosis to disease-modifying therapies. Postmortem analysis of brains from PD patients reveals a substantial decrease in SN neurons within the initial four years post-diagnosis (50-90% decrease), rendering symptomatic treatments less effective at the diagnosed disease stage (136). The identification of immunologic biomarkers holds promise for early disease detection and thus, enabling earlier and presumably more effective interventions. An interesting epidemiological study revealed that individuals with pre-existing Inflammatory Bowel Disease (IBD) have a higher risk of developing PD. However, exposure to anti-TNF therapy conferred protection against PD (137), providing further proof of an immune component in PD, and strongly suggest possibilities to identify and treat high-risk patients very early. Additionally, we recently demonstrated in a longitudinal case study that α-syn specific T cells were present more than a decade before the occurrence of PD symptoms, suggesting avenues in potential early diagnosis (76). Early diagnosis could also be based on the identification of markers of disease-associated gut dysbiosis. In fact, gut dysbiosis may occur years before motor symptoms in PD and MSA, suggesting that specific microbiota signatures may yield predictive biomarkers for early diagnosis (41). An important consideration to identify robust marker(s) is to take into account the biological sex, as we have reviewed here, many differences are specific to males or females.

Targeting inflammation to delay, halt or reverse the immune response represents a promising strategy, drawing from extensive research in other autoimmune/inflammatory contexts. Clinical trials testing drugs aimed at reducing microglial activation and inflammation, such as verdiperstat (BHV-3241), are underway in MSA patients (NCT04616456). Cell-based approaches, like intravenous allogeneic bone marrow-derived mesenchymal stem cell (MSC) therapy, demonstrate neuroprotective effects through anti-inflammatory actions mediated by microglial activation modulation (138), though long-term safety and clinical benefits require further investigation. Myeloid cells such as monocytes, macrophages and BAM can also be targeted. However, drugs need to be specific as in MSA, the use of an CSF1R inhibitor showed dual results both beneficial on the lifespan but deleterious on motor symptoms (106, 107). Blocking the entry of monocytes into the CNS via targeting CCL2 (113) represents a potential area.

Several clinical trials aim to modulate the immune response through lymphocytes and their mediators (139). For instance, anti-CD3 monoclonal antibodies (mAbs) therapies trigger apoptosis in activated T cells and spares Tregs, thus preventing the exacerbated T-cell mediated inflammation. However, while some trials have shown promising results in other diseases, others have raised safety concerns (140). Another therapeutic strategy could be based on B cell depletion therapies like rituximab and ocrelizumab, which target CD20-expressing B cells and have been found to be successful in other CNS diseases such as multiple sclerosis (141). Therefore, anti-CD20 therapies could offer an interesting therapeutic perspective to prevent B-cell mediated inflammation in synucleinopathies. However, B cells depleting therapies are not yet supported by the literature as the role of B cells in synucleinopathies is still not fully understood.

Furthermore, in view of the emerging gut-brain theory, clinical trials also try to target inflammation in the gut. Among them, a clinical trial ongoing assess the efficiency of fecal microbiota transplantation to restore gut homeostasis and reduce inflammation on PD patients (NCT03808389), in line with *in vivo* transplantation experiments (37). Additional resources are also available for further information on clinical trials, including those not specifically targeting inflammation (142).

## 7 Conclusion

The understanding of synucleinopathies has evolved significantly, shedding light on the complexities of disease initiation, progression, and the pivotal role of inflammation in the pathogenesis. The body-first and brain-first subtypes offer distinct trajectories in disease onset and progression, emphasizing the need for targeted therapeutic approaches. The body-first hypothesis implicates the enteric nervous system and gut-brain axis, highlighting the role of microbial dysbiosis and inflammatory responses in α-syn aggregation and propagation. The gut being the largest interface with the environment, it is highly thought to be the pathway to environmental stressors. Indeed, numerous by-products of the industrial revolution, including specific pesticides and heavy metals, have been linked to increased cases of PD (143). Recent studies showed that adherence to Mediterranean diet was associated with a lower risk of Alzheimer’s disease and PD development thanks to a beneficial microbial composition that reduces the risk of inflammation (144). Conversely, the brain-first hypothesis suggests a primary CNS origin of pathology, with differential involvement of resident cells such as microglia and astrocytes in initiating and perpetuating neuroinflammation.

Microglia and astrocytes, traditionally recognized for their homeostatic roles, are now acknowledged as key players in neuroinflammation. Microglia-mediated inflammatory responses, triggered by α-syn aggregates, contribute to neuronal degeneration, facilitating peripheral immune cell infiltration. Astrocytes transit into a neurotoxic A1 phenotype in response to α-syn, exacerbating inflammation and synaptic degeneration. The involvement of peripheral immune cells, particularly T and B cells, further complicates the inflammatory landscape of synucleinopathies, with implications for both adaptive and innate immunity. Sex-based differences add another layer of complexity to synucleinopathies, influencing disease prevalence, phenotype, and immune responses. Biological sex affects neuroinflammatory pathways and the immune response, suggesting the need for sex-specific therapeutic strategies and biomarker identification. However, the involvement of the immune system, the autoimmune component and sex-based differences provide new promising therapeutic avenues. Indeed, those strategies and differences have been more intensively studied and developed in other diseases (145), eventually helping us to better understand and design therapies.

Unraveling the intricate interplay between inflammation, immune responses, and disease progression in synucleinopathies holds immense promise for developing effective treatments and advancing personalized medicine approaches tailored to the distinct subtypes and individual characteristics of patients.
